# The sudden death and sudden birth of quantum discord

**DOI:** 10.1038/s41598-018-23639-1

**Published:** 2018-03-28

**Authors:** Wei Xia, Jin-Xing Hou, Xiao-Hui Wang, Si-Yuan Liu

**Affiliations:** 1 Institute of Modern Physics, Northwest University, Xi’an, 710069 China; 2 School of Physics, Northwest University, Xi’an, 710069 China; 3 Shaanxi Key Laboratory for Theoretical Physics Frontiers, Xi’an, 710069 China; 4 Institute of Physics, Chinese Academy of Sciences, Beijing, 100190 China

## Abstract

The interaction of quantum system and its environment brings out abundant quantum phenomenons. The sudden death of quantum resources, including entanglement, quantum discord and coherence, have been studied from the perspective of quantum breaking channels (QBC). QBC of quantum resources reveal the common features of quantum resources. The definition of QBC implies the relationship between quantum resources. However, sudden death of quantum resources can also appear under some other quantum channels. We consider the dynamics of Bell-diagonal states under a stochastic dephasing noise along the *z*-direction, and the sudden death and sudden birth of quantum discord are investigated. Next we explain this phenomenon from the geometric structure of quantum discord. According to the above results, the states with sudden death and sudden birth can be filtered in three-parameter space. Then we provide two necessary conditions to judge which kind of noise channels can make Bell-diagonal states sudden death and sudden birth. Moreover, the relation between quantum discord and coherence indicates that the sudden death and sudden birth of quantum discord implies the sudden death and sudden birth of coherence in an optimal basis.

## Introduction

Quantum resource theories^1,2^ have played a potential role in understanding various physical phenomenons in quantum physics and quantum information theory. A resource theory consists of two basic elements: free operations and free states. Quantum correlations, including entanglement, quantum discord and quantum coherence, are regarded as crucial resources in quantum information processing^3–16^. It is stipulated that separable states, classical correlated states and incoherent states are the free states of entanglement, quantum discord and quantum coherence, respectively.

In recent years, the behavior of quantum resources derived from the interaction of quantum system and its environment, such as sudden death, has attracted a great deal of attention. The sudden death of quantum resources means that the resource states convert into free states under quantum channels within a finite time. Sudden death of quantum resources, which should be avoided in quantum information processing, has been widely studied both theoretically and experimentally^17–19^. The sudden death phenomenon has been investigated from the perspective of constructing resources breaking channels in recent years. The hierarchy of quantum resources has been clearly researched^20^, and the relationships between quantum resources have been studied in many aspects^20–24^. The breaking channels give a method to vanish the quantum resources, and reveal the common features of quantum resources. In this work, we investigate the hierarchy of quantum resources breaking channels, and try to give a unified view of quantum resources.

It is conventional that the quantum noise channels vanish the quantum resources and convert the resources states into free states. A natural question is that whether the free states can convert into resources states under quantum channels, i.e., the sudden birth of quantum resources. In this paper, we consider the case that two-qubit system globally interacting with stochastic dephasing noise along the *z*-direction. It is shown that quantum discord sudden death and sudden birth at time *t*_*db*_. The sudden birth indicates that quantum discord can be created by quantum noise channels. Latterly, some researchers have point out that quantum discord is equal to coherence in a set of mutually unbiased bases for Bell-diagonal states^25^. Naturally, the sudden death and sudden birth of quantum discord can be regarded as sudden death and sudden birth of quantum coherence in an optimal basis.

Two-qubit Bell-diagonal states, which can be depicted as a tetrahedron in three dimensions, are significant for understanding states with more complex structure. For Bell-diagonal states, the geometric structure of quantum resources shows the states with zero resources (free states) intuitively. It is known that the initial states and quantum channels determine the trajectory of the states evolution. The trajectory approaches to the free states in a finite time means the sudden death of quantum resources. In this paper, we would like to study the sudden death and sudden birth of quantum resources geometrically and propose two necessary conditions to judge which quantum noise channels have this interesting phenomenon for Bell-diagonal states.

This paper is arranged as follows. We start with a brief introduction of quantum correlations and dynamics of open quantum systems. Next we indicate the unified expression of breaking channels and give the hierarchical relationship of corresponding free states. For Bell-diagonal states, we introduce the model and investigate the sudden death and sudden birth of quantum correlations under a stochastic dephasing noise along the *z*-direction and we give a brief and clear explanation from geometric point of view and extract the states with this interesting phenomenon. Then, two necessary conditions are presented under some noise channels, which help us to understand the sudden death and sudden birth of Bell-diagonal states. In addition, we propose that sudden death and sudden birth of quantum discord can be identified as sudden death and sudden birth of quantum coherence in an optimal basis. Finally, we summarize the conclusions.

## Review of Relevant Definition

### Review of quantum discord

The quantum mutual information of system *A* and *B* is given by

1 $${\mathscr{I}}(A:B)=S(A)+S(B)-S(AB).$$

And the classical mutual information has the following form^26^

2 $${{\mathscr{J}}}_{cl}(A|B)=S(B)-S(B|A),$$

where $S(B|A)={\sum }_{a}\,{p}_{a}S(B|a)$ is the conditional entropy^27^. It allows us to introduce classical correlation of the state *ρ*_*AB*_ in analogy with Eq. (2) ^28^

3 $${\mathscr{J}}(B|\{{{\rm{\Pi }}}_{a}\})=S(B)-S(B|\{{{\rm{\Pi }}}_{a}\}),$$

where {Π_*a*_} is the set of von Neumann measurements with classical outcome *a* on subsystem *A*. The quantum discord of a state *ρ*_*AB*_^29^ is defined as the difference between total correlations, as given by the quantum mutual information Eq. (1), and the classical correlation (3)

4 $${\mathscr{D}}(B|A)\equiv \mathop{{\rm{\min }}}\limits_{{{\rm{\Pi }}}_{a}}\,\{ {\mathcal I} (A:B)-{\mathscr{J}}(B|\{{{\rm{\Pi }}}_{a}\})\},$$

where ${\mathscr{D}}(B|A)$ is minimized over the sets of von Neumann measurements.

### Dynamics of open quantum system

The time evolution of a general closed quantum system is described by the Liouville-von Neumann equation (which takes *ħ* = 1)

5 $$\dot{\rho }(t)=-i[ {\mathcal H} ,\rho (t)],$$

where *ρ* and ${\mathcal H}$ are the density operator and Hamiltonian of the system, respectively. However, the realistic system (*S*) always interacts with its surrounding environment (*E*). Considering the interaction between system and its environment, the complete Hamiltonian can be written as

6 $$ {\mathcal H} ={ {\mathcal H} }_{S}+{ {\mathcal H} }_{E}+{ {\mathcal H} }_{I},$$

where ${ {\mathcal H} }_{S}$ and ${ {\mathcal H} }_{E}$ are the system and environment Hamiltonian, ${ {\mathcal H} }_{I}$ is the interaction Hamiltonian. The dynamics of the whole system (*S* + *E*) is governed by master equation with total Hamiltonian ${\mathcal H}$. The dynamics of the reduced system (*S*) traced out the environment (*E*) can be expressed as^30^

7 $${\dot{\rho }}_{S}(t)=-iT{r}_{E}[ {\mathcal H} ,{\rho }_{SE}(t\mathrm{)]}.$$

We can use the master equation approach to solve Eq. (7), but there is a more appropriate way to realize our purposes, which is known as operator-sum representation. The general solution of Eq. (7) can be written as

8 $${\rho }_{SE}(t)=U(t){\rho }_{SE}{U}^{\dagger }(t),$$

where *U*(*t*) is the unitary evolution operator generated by the total (*S* + *E*) Hamiltonian. The description of system (*S*) evolution under the action of the environment *E* is given by

9 $$\varepsilon ({\rho }_{S})=T{r}_{E}[U(t){\rho }_{SE}{U}^{\dagger }(t\mathrm{)]}.$$

The map *ε* is a quantum operation and *ε*(*ρ*_*s*_) represents a final state. Denoting {|*e*_*i*_〉} is a set of orthonormal basis for the state space of environment, and ${\rho }_{E}=|{e}_{0}\rangle \langle {e}_{0}|$ is the initial state of environment. And then, Eq. (9) can be expressed as the form of operator-sum representation

10 $$\varepsilon ({\rho }_{S})=\sum _{i}\,\langle {e}_{i}|U(t)\,[{\rho }_{S}\otimes |{e}_{0}\rangle \,\langle {e}_{0}|]U{(t)}^{\dagger }|{e}_{i}\rangle =\sum _{i}\,{K}_{i}{\rho }_{S}{K}_{i}^{\dagger },$$

with the Kraus operator^27^ ${K}_{i}\equiv \langle {e}_{i}|U(t)|{e}_{0}\rangle$. A map *ε*, which is called a quantum channel, is completely positive trace preserving (CPTP) with the constrain ${\sum }_{i}\,|{K}_{i}\rangle \langle {K}_{i}|={\bf{I}}$. For many-body system, two kinds of environments should be considered: (i) global and (ii) local environment. In case (i), the interaction of all parts of *S* with the same environment may increase the correlations between the parts of system due to nonlocal interactions mediated by the environment. In case (ii), each part of system *S* interacts with their own local and independent environment, the effects of environment can not increase the correlations between the parts of system. Detailed description will be introduced in the later.

### Review of quantum coherence

Quantum coherence, a fundamental property of quantum mechanics, marks the departure of quantum physics from classical physics. A reasonable measure of quantum coherence should fulfill the following conditions^31^

**Nonnegativity** ${\mathscr{C}}(\rho )\ge 0$ with equality if and only if *ρ* is an incoherent states;

**Monotonicity** ${\mathscr{C}}$ do not increase under the action of incoherent operations, ${\mathscr{C}}({\rm{\Lambda }}[\rho ])\le {\mathscr{C}}(\rho )$, for any incoherent operation Λ;

**Strong monotonicity** ${\mathscr{C}}$ do not increase on average under selective incoherence operations, ${\sum }_{i}\,{q}_{i}{\mathscr{C}}({\rho }_{i})\le {\mathscr{C}}(\rho )$, with probabilities ${q}_{i}=Tr[{K}_{i}\rho {K}_{i}^{\dagger }]$, ${\rho }_{i}={K}_{i}\rho {K}_{i}^{\dagger }/{q}_{i}$, and incoherent Kraus operators *K*_*i*_;

**Convexity** ${\mathscr{C}}$ is a convex function of the state, ${\sum }_{i}\,{p}_{i}{\mathscr{C}}({\rho }_{i})\ge {\mathscr{C}}(\sum \,{p}_{i}{\rho }_{i})$.

Based on the framework of quantum coherence, the relative entropy of quantum coherence is defined as

11 $${{\mathscr{C}}}_{re}(\rho )=\mathop{{\rm{\min }}}\limits_{\delta \in {\mathbb{I}}}\,S(\rho \parallel \delta )=S({\rho }_{diag})-S(\rho ),$$

where *ρ*_*diag*_ comes from *ρ* by dropping off-diagonal elements and ${\mathbb{I}}$ represents the set of incoherent states, $S(\rho \parallel \delta )={\rm{Tr}}(\rho \,\mathrm{log}\,\rho -\rho \,\mathrm{log}\,\delta )$ is the quantum relative entropy^27^ and $S(\rho )=-{\rm{Tr}}(\rho \,\mathrm{log}\,\rho )$ is the von Neumann entropy.

## Breaking channels and sudden death of quantum resources

Any quantum channel on a single quantum system will be called a breaking channel if it maps any state to the corresponding free state. Holevo introduces a special class of stochastic maps^32^, as the following form:

12 $${\rm{\Phi }}(\rho )=\sum _{k}\,{R}_{k}Tr({F}_{k}\rho ),$$

where each *R*_*k*_ is a density matrix and the {*F*_*k*_} form a positive operator valued measure (POVM). Shor then proved that a channel can be written as Eq. (12) if and only if it is entanglement breaking^33^, so this kind of channel is called entanglement breaking channels (EBC). If each ${F}_{k}=|k\rangle \langle k|$ in the POVM is a one-dimension projection, this kind of channel is called classical-quantum (CQ) channels. In this case, the Holevo form reduces to ${\rm{\Phi }}(\rho )={\sum }_{k}\,{R}_{k}\langle k|\rho |k\rangle$. When each density matrix ${R}_{k}=|k\rangle \langle k|$ is one-dimensional projection and ${\sum }_{k}\,{R}_{k}={\bf{I}}$, this kind of channel is called quantum-classical (QC) channels. In terms of the definition of CQ and QC channels, we assume that there are two qubits *A* and *B* with non-zero discord. After applying the QC channels to subsystem *B*, the dynamics of the system is

13 $$({\bf{I}}\otimes {{\rm{\Phi }}}_{QC}){\rho }_{AB}=\sum _{k}\,|k\rangle \langle k|\otimes T{r}_{B}({F}_{k}{\rho }_{AB}).$$

It is shown that *B* becomes a classical state and *A* is still a quantum state. Analogously, after applying the CQ channels to subsystem *B*, it’s easy to see that the dynamics of the system is

14 $$({\bf{I}}\otimes {{\rm{\Phi }}}_{CQ}){\rho }_{AB}=\sum _{k}\,{R}_{k}\otimes T{r}_{B}(|k\rangle \langle k|{\rho }_{AB})=\sum _{k}\,{R}_{k}\otimes \langle k|{\rho }_{AB}|k\rangle .$$

**Figure 1:**
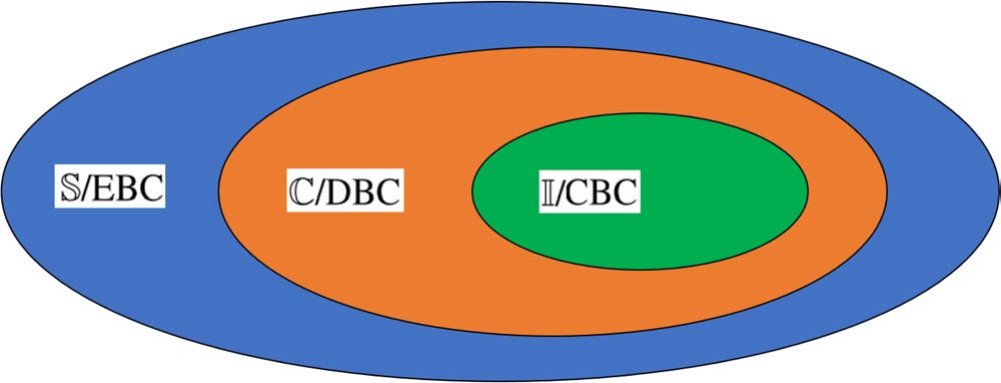
The blue region, orange region and green region stand for the sets of ${\mathbb{S}}$/EBC, ${\mathbb{C}}$/DBC and ${\mathbb{I}}$/CBC respectively, where ${\mathbb{S}}$ and ${\mathbb{C}}$ stand for separable states and classical correlated states.

Obviously, *A* converts into a classical state and *B* is still a quantum state. Since QC channels and CQ channels map quantum states to classical correlated states, they are called discord breaking channels (DBC). A coherence breaking channel (CBC) is a QC channel, which has been proved in ref.^34^. Therefore, we obtain a conclusion that three kinds of breaking channels have a unified mathematical expression called the Holevo form. The hierarchical relationship of breaking channels^34^ and free states can be directly obtained, as shown in Fig. 1

According to the above results, we know very well that the resourceful states can be mapped to free states by corresponding breaking channels, and the sudden death appears during the process. In this paper, we study the same phenomenon under both the local and collective dephasing channels. Unexpectedly, we find the sudden death and sudden birth of quantum discord, we will introduce it in next section.

## The sudden death and sudden birth of quantum discord

### The model

**Figure 2:**
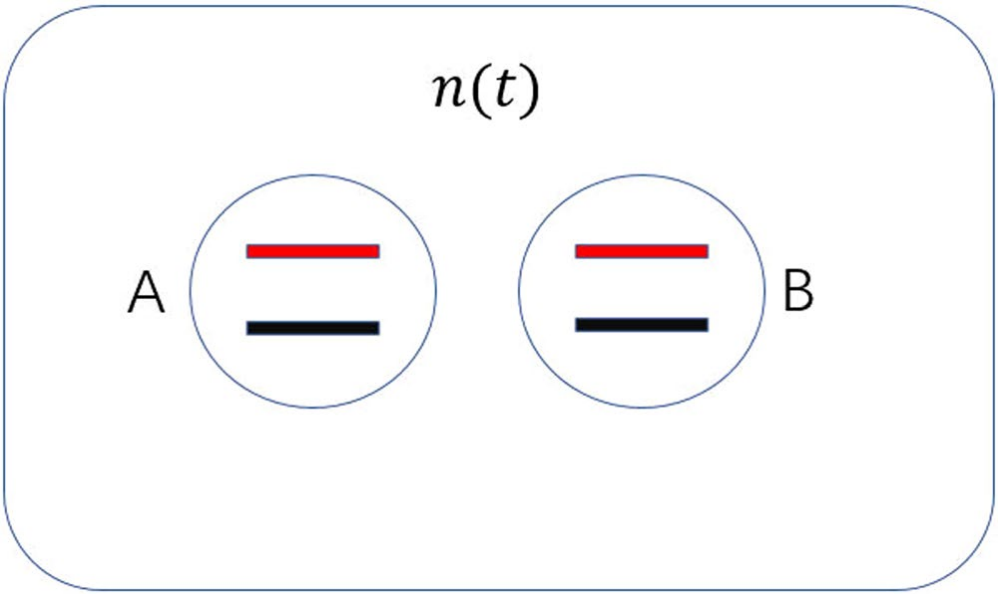
The model consists of two qubits *A* and *B* interacting with the stochastic field *n*(*t*), and *A* and *B* are two-level energy systems. The initial states of system *A* and *B* is not coupled with the environment.

A two-qubit system coupled to a noisy environment collectively is considered in this paper, which is shown in Fig. 2. The system can be described by following Hamiltonian^35^ (which takes *ħ* = 1 and adopts spin notation):

15 $$ {\mathcal H} (t)=-\frac{1}{2}n(t)\,({\sigma }_{z}^{A}\otimes {{\bf{I}}}^{B}+{{\bf{I}}}^{A}\otimes {\sigma }_{z}^{B}),$$

where *n*(*t*) is a stochastic field with $\langle n(t)\rangle =0\,\& \,\langle n(t)n(t^{\prime} )\rangle ={\rm{\Gamma }}\delta (t-t^{\prime} )$, Γ is the damping rate associated with the field *n*(*t*), and ${\sigma }_{z}^{A,B}$ are the Pauli matrices

16 $${\sigma }_{z}^{A,B}=(\begin{array}{cc}1 & 0\\ 0 & -1\end{array}).$$

The dynamics under the Hamiltonian Eq. (15) can be described by a master equation or a set of Kraus operators. In this paper, we consider the latter. It’s known that quantum channels *ε* can be characterized by a set of Kraus operators *K*_*i*_. For any initial state, the action of quantum map *ε* is given by

17 $$\rho (t)=\varepsilon (\rho \mathrm{(0))}=\sum _{i=1}^{N}\,{K}_{i}^{\dagger }(t)\rho \mathrm{(0)}{K}_{i}(t),$$

the operators *K*_*i*_ contain all the information about the system’s dynamics. The most general solution can be expressed in terms of three Kraus operators (under the assumption that the initial density matrix is not correlated with any of environments)^36^. The Kraus operators which describe the interaction with the environmental fields are given by

18 $${K}_{1}=(\begin{array}{cccc}\gamma (t) & 0 & 0 & 0\\ 0 & 1 & 0 & 0\\ 0 & 0 & 1 & 0\\ 0 & 0 & 0 & \gamma (t)\end{array}),$$

19 $${K}_{2}=(\begin{array}{cccc}{\omega }_{1}(t) & 0 & 0 & 0\\ 0 & 0 & 0 & 0\\ 0 & 0 & 0 & 0\\ 0 & 0 & 0 & {\omega }_{2}(t)\end{array}),$$

20 $${K}_{3}=(\begin{array}{cccc}0 & 0 & 0 & 0\\ 0 & 0 & 0 & 0\\ 0 & 0 & 0 & 0\\ 0 & 0 & 0 & {\omega }_{3}(t)\end{array}).$$

This channel is known as stochastic dephasing channel along the *z*-direction^35^. Note that the parameters are given by

21 $$\{\begin{array}{ccc}\gamma (t) & = & {e}^{-t/2T},{\omega }_{1}(t)=\sqrt{1-{e}^{-t/T}},\\ {\omega }_{2}(t) & = & -{e}^{-t/T}\sqrt{1-{e}^{-t/T}},\\ {\omega }_{3}(t) & = & \sqrt{(1-{e}^{-t/T})\,(1-{e}^{-2t/T}),}\end{array}$$

where *T* = 1/Γ is the phase relaxation time due to the collective interaction with *n*(*t*).

### Sudden death and sudden birth of quantum discord

**Figure 3:**
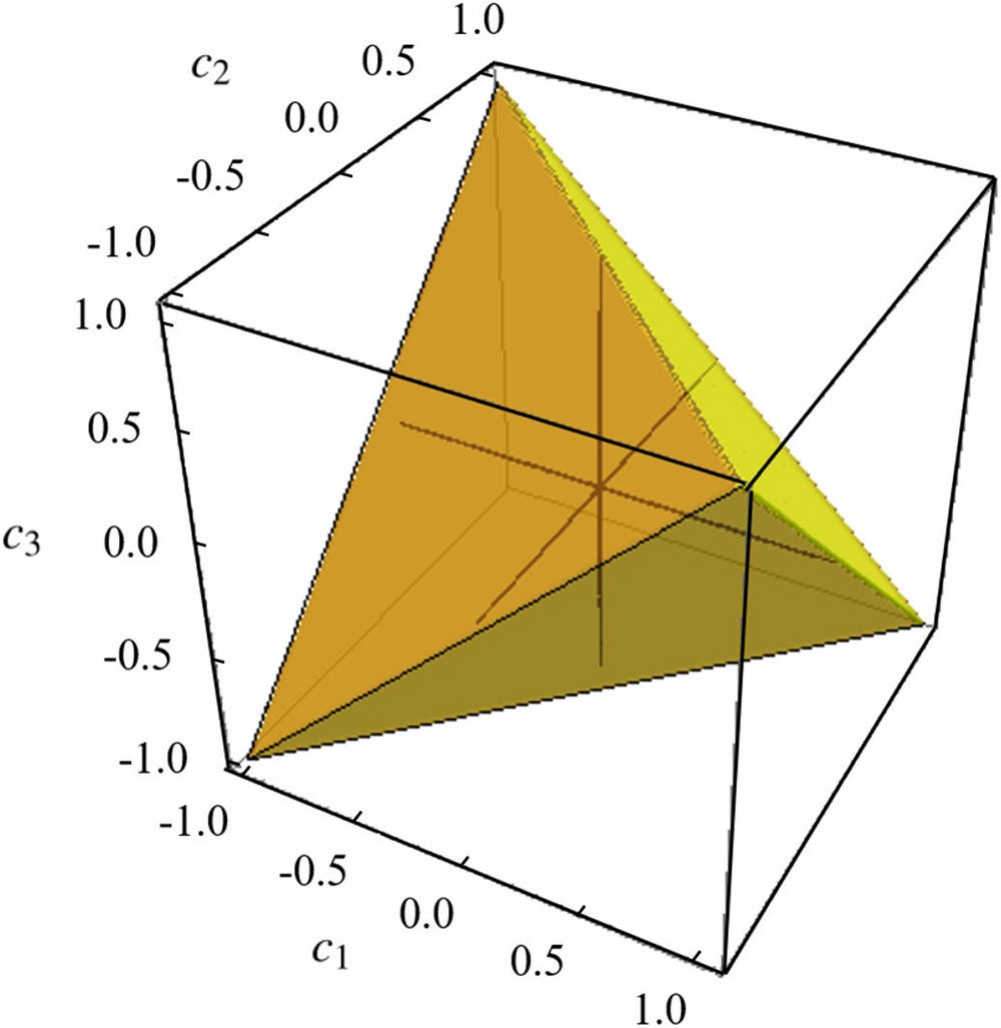
Two-qubit Bell-diagonal states described by parameters *c*_1_, *c*_2_ and *c*_3_ can be depicted as a regular tetrahedron. The black lines in tetrahedron are coordinate axes in which the states have zero quantum discord.

Two-qubit Bell-diagonal states with the maximally mixed reduced density matrix are given by

22 $${\rho }_{AB}=\frac{1}{4}({\bf{I}}\otimes {\bf{I}}+\sum _{j=1}^{3}\,{c}_{j}{\sigma }_{j}\otimes {\sigma }_{j}),$$

where *σ*_*j*_ are Pauli operators, *c*_*j*_ are real numbers such that 0 ≤ |*c*_*j*_| ≤ 1. They constitute a tetrahedron in parameter space as shown in Fig. 3. The eigenstates of *ρ*_*AB*_ are the four Bell states

23 $$|{{\rm{\Phi }}}_{AB}\rangle \equiv \mathrm{(|0},b\rangle +{(-\mathrm{1)}}^{a}\mathrm{|1},1\oplus b\rangle )\sqrt{2},$$

with eigenvalues

24 $${\lambda }_{ab}=\frac{1}{4}\mathrm{[1}+{(-\mathrm{1)}}^{a}{c}_{1}-{(-\mathrm{1)}}^{a+b}{c}_{2}+{(-\mathrm{1)}}^{b}{c}_{3}],$$

where *a* ∈ (0, 1), *b* ∈ (0, 1), *λ*_*ab*_ ∈ [0, 1]. For two-qubit Bell-diagonal states, the quantum discord is given by^37^

25 $${\mathscr{D}}({\rho }_{AB})=-H({\lambda }_{ab})-\sum _{j=1}^{2}\,\frac{\mathrm{(1}+{(-\mathrm{1)}}^{j}c)}{2}\,{\mathrm{log}}_{2}\,\frac{\mathrm{(1}+{(-\mathrm{1)}}^{j}c)}{4},$$

where *c* = max{|*c*_1_|, |*c*_2_|, |*c*_3_|} and $H({\lambda }_{ab})=-{\sum }_{a,b}\,{\lambda }_{ab}\,\mathrm{log}\,{\lambda }_{ab}$ is the Shannon entropy^38^. The relative entropy of coherence ${{\mathscr{C}}}_{re}$ is given by

26 $${{\mathscr{C}}}_{re}({\rho }_{AB})=-H({\lambda }_{ab})-\sum _{j=1}^{2}\,\frac{\mathrm{(1}+{(-\mathrm{1)}}^{j}{c}_{3})}{2}\,{\mathrm{log}}_{2}\,\frac{\mathrm{(1}+{(-\mathrm{1)}}^{j}{c}_{3})}{4}.$$

The classical correlation (${\mathscr{C}}{\mathscr{C}}$) equals to^37^

27 $${\mathscr{C}}{\mathscr{C}}=1-H(\frac{1+c}{2})=\frac{1+c}{2}\,{\mathrm{log}}_{2}\,\mathrm{(1}+c)+\frac{1-c}{2}\,{\mathrm{log}}_{2}\mathrm{(1}-c),$$

Interacting with the stochastic dephasing channels, *ρ*_*AB*_(*t*) is given by

28 $${\rho }_{AB}(t)=(\begin{array}{cccc}1+{c}_{3} & 0 & 0 & {\gamma }^{4}({c}_{1}-{c}_{2})\\ 0 & 1-{c}_{3} & {c}_{1}+{c}_{2} & 0\\ 0 & {c}_{1}+{c}_{2} & 1-{c}_{3} & 0\\ {\gamma }^{4}({c}_{1}-{c}_{2}) & 0 & 0 & 1+{c}_{3}\end{array}).$$

It is obvious that the quantum channels make *c*_3_ and *c*_1_ + *c*_2_ invariant, while *c*_1_ − *c*_2_ is exponential decay. The dynamics of *ρ*_*AB*_(*t*) can be simply expressed as

29 $$\{\begin{array}{ccc}{c}_{1}(t) & = & \frac{1}{2}[{c}_{1}(1+{e}^{-2{\rm{\Gamma }}t})+{c}_{2}(1-{e}^{-2{\rm{\Gamma }}t})],\\ {c}_{2}(t) & = & \frac{1}{2}[{c}_{1}(1-{e}^{-2{\rm{\Gamma }}t})+{c}_{2}(1+{e}^{-2{\rm{\Gamma }}t})],\\ {c}_{3}(t) & = & {c}_{3}(0).\end{array}$$

We also consider the two-qubit local dephasing noises and single qubit local dephasing noises, however, the quantum discord don’t have sudden death and sudden birth under those two kinds of local noises and their parameters satisfy the following mathematic relations^36^:

30 $$\{\begin{array}{ccc}{c}_{1}(t) & = & 2{c}_{1}{\gamma }_{A}{\gamma }_{B},\\ {c}_{2}(t) & = & 2{c}_{2}{\gamma }_{A}{\gamma }_{B},\\ {c}_{3}(t) & = & {c}_{3}(0),\end{array}$$

31 $$\{\begin{array}{ccc}{c}_{1}(t) & = & 2{c}_{1}{\gamma }_{A},\\ {c}_{2}(t) & = & 2{c}_{2}{\gamma }_{A},\\ {c}_{3}(t) & = & {c}_{3}(0),\end{array}$$

where ${\gamma }_{A}={e}^{-t\mathrm{/2}{T}_{A}^{2}}$, ${\gamma }_{B}={e}^{-t\mathrm{/2}{T}_{B}^{2}}$. ${T}_{A}^{2}=\mathrm{1/}{{\rm{\Gamma }}}_{A}$ and ${T}_{B}^{2}=\mathrm{1/}{{\rm{\Gamma }}}_{B}$ are the phase relaxation time for qubit *A* and qubit *B* due to the interaction with their own environment. With this in mind, it is verified that the collective quantum channels can enhance the correlations between the parts of system, while local channels can not. One of necessary conditions can explain that and a more detailed description is described below.

**Figure 4:**
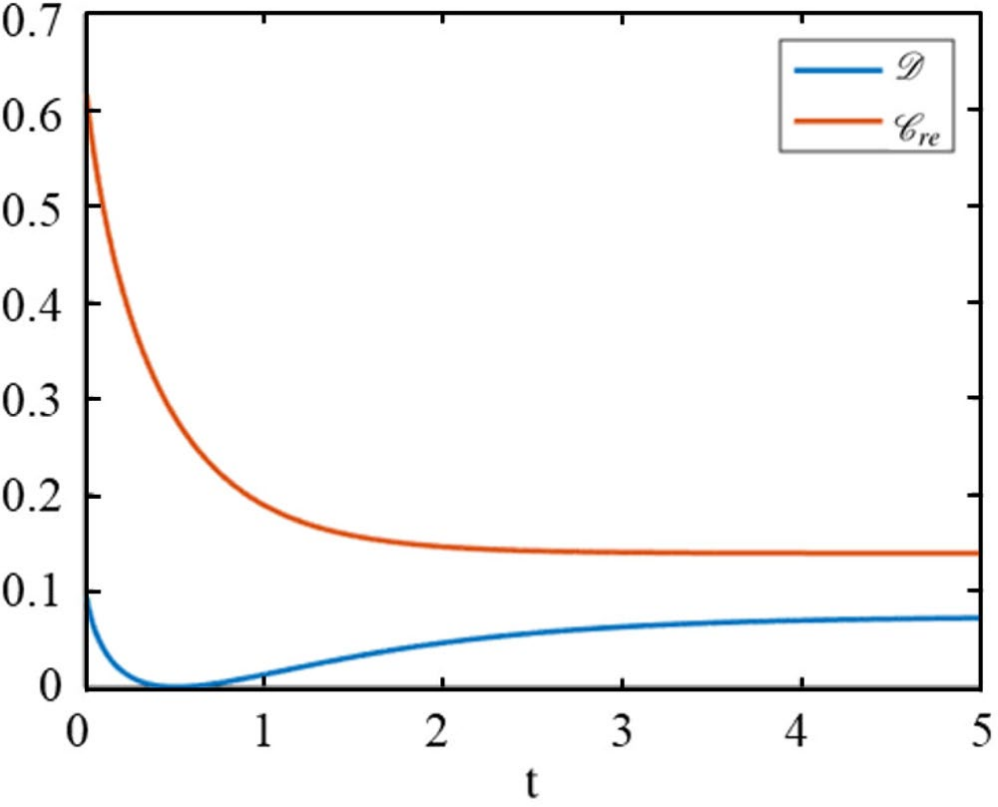
The orange line is coherence and the blue line is quantum discord. For initial state *c*_1_ = 0.8, *c*_2_ = −0.2, *c*_3_ = 0, Γ = 0.5, the sudden death and sudden birth of quantum discord appear at time *t* = 0.510, which signifies *c*_2_(*t*) = 0.

The dynamics of quantum discord and coherence under global stochastic dephasing channel is depicted in Fig. 4. It is clear that the coherence decreases monotonously to 0.139 with time *t*. However, quantum discord decreases to zero in a finite time *t* = 0.510 and increase from zero to a limited value for the time *t* > 0.510, as $t\to \infty$, ${\mathscr{D}}\to 0.072$. It reveals the sudden death and sudden birth phenomenon of quantum discord.

**Figure 5:**
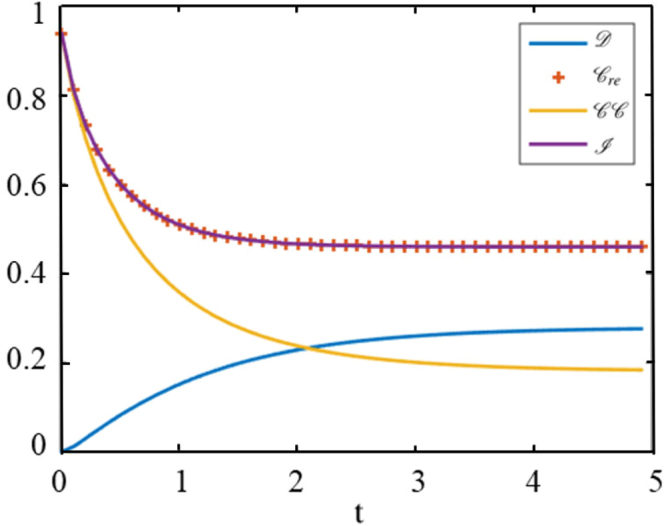
${\mathscr{D}}$, ${{\mathscr{C}}}_{re}$, ${\mathscr{C}}{\mathscr{C}}$ and ${\mathcal I}$ are quantum discord, the relative entropy of quantum coherence, classical correlation and mutual information, respectively. For initial state *c*_1_ = 0.99, *c*_2_ = −0.01, *c*_3_ = 0, Γ = 0.5, quantum discord appears sudden death and sudden birth at *t* = 0.02.

Figure 5 shows that the mutual information (${\mathcal I}$) and classical correlation (${\mathscr{C}}{\mathscr{C}}$) decrease with *t*, ${\mathcal I}$ and ${{\mathscr{C}}}_{re}$ are equivalent. This phenomenon comes from the following mathematic expression:

32 $$\begin{array}{rcl}{{\mathscr{C}}}_{re} & = & -H({\lambda }_{ab})+\mathrm{2,}\\  {\mathcal I}  & = & {\mathscr{D}}+{\mathscr{C}}{\mathscr{C}}=-H({\lambda }_{ab})-\sum _{j=1}^{2}\,\tfrac{\mathrm{(1}+{(-\mathrm{1)}}^{j}c)}{2}\,{\mathrm{log}}_{2}\,\tfrac{\mathrm{(1}+{(-\mathrm{1)}}^{j}c)}{4}+\sum _{j=1}^{2}\,\tfrac{\mathrm{(1}+{(-\mathrm{1)}}^{j}c)}{2}\,{\mathrm{log}}_{2}\,\mathrm{(1}+{(-\mathrm{1)}}^{j}c),\\  & = & -H({\lambda }_{ab})+2.\end{array}$$

It’s necessary to point out that discord still have the sudden death and sudden birth phenomenon in the picture though the transition point very closes to zero. After sudden death of discord, ${\mathcal I}$ and ${{\mathscr{C}}}_{re}$ tend to be gradually invariable. ${\mathscr{C}}{\mathscr{C}}$ declines but ${\mathscr{D}}$ increases, which signifies that the price of discord’s sudden birth is the consumption of classical correlation. In other words, classical correlation can convert into quantum discord.

## Discussion for the Dynamics of Quantum States

In this subsection, we are going to investigate the sudden death and sudden birth of quantum discord from the geometric structure of quantum discord. For two-qubit Bell-diagonal states, quantum discord is zero if and only if the states are limited to axes. It is known that the dynamic trajectory is determined by the quantum channels and initial states. Sudden death and sudden birth of quantum discord means that the dynamic trajectory passes through coordinate axes.

**Figure 6:**
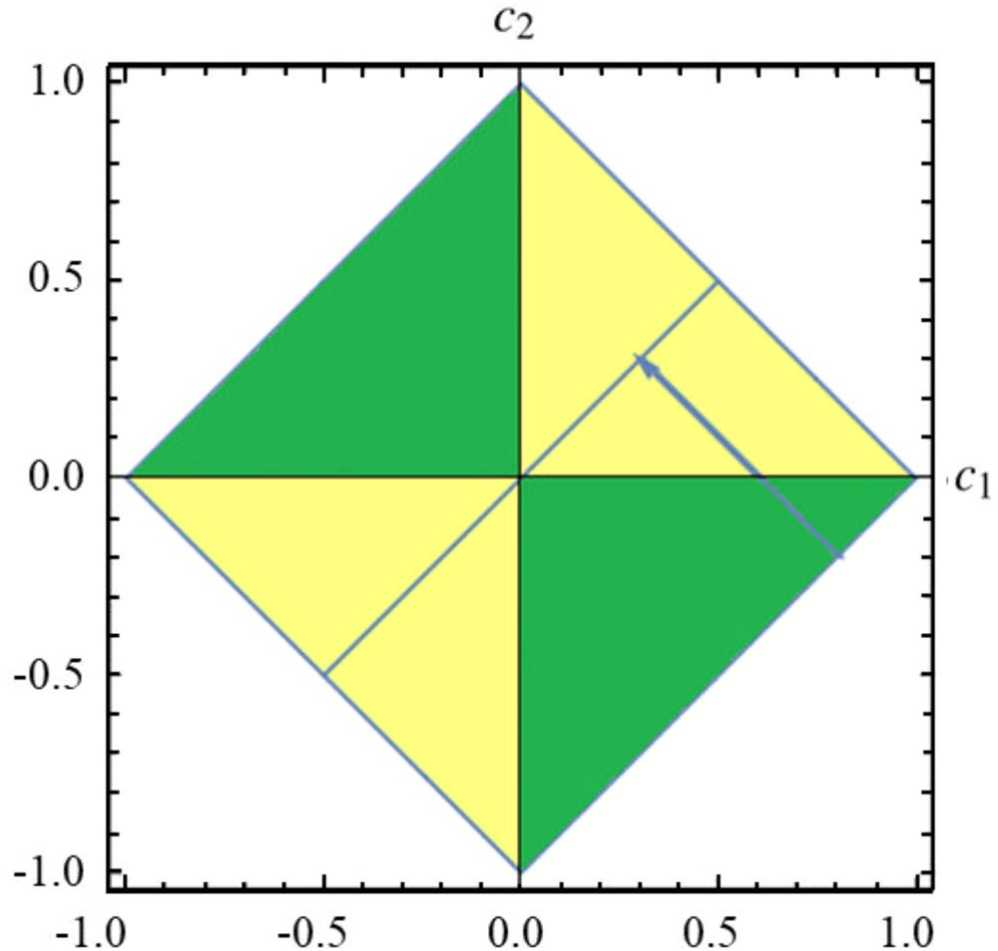
The quadrangle is *c*_3_ = 0 section of regular tetrahedron and blue line represents *c*_1_ = *c*_2_. The parameters of initial state are *c*_1_ = 0.8, *c*_2_ = −0.2, Γ = 0.5. According to Eq. (29), we know *c*_1_(*t*) + *c*_2_(*t*) = *c*_1_ + *c*_2_ and *c*_1_(*t*) − *c*_2_(*t*) = (*c*_1_ − *c*_2_) *e*^−2Γ*t*^. Therefore, the arrowed line which represents evolutionary track of initial state is *c*_2_(*t*) = −*c*_1_(*t*) + 0.6 and end point is *c*_1_ = *c*_2_ = 0.3. The states in the green region represent that they have sudden death and sudden birth, because if the initial states are chosen in this region, their evolutionary track are perpendicular to the blue line, namely, they pass the *c*_1_ axis.

In Fig. 6, the dynamic trajectory of quantum states under global stochastic dephasing channel is a straight line towards the plane *c*_1_ = *c*_3_, and it obviously passes though the *c*_2_ axis, namely, discord could experience the sudden death and sudden birth. For the initial states (*c*_1_(0), *c*_2_(0), *c*_3_(0)) limited to regions *c*_2_ − *c*_1_ ≤ 1, *c*_1_ < 0, *c*_2_ > 0 and *c*_2_ − *c*_1_ ≥ −1, *c*_1_ > 0, *c*_2_ < 0, quantum discord has the phenomenon of sudden death and sudden birth at time ${t}_{db}=\frac{1}{2{\rm{\Gamma }}}\,\mathrm{ln}\,\frac{{c}_{1}-{c}_{2}}{{c}_{1}+{c}_{2}}$. The dynamic trajectory of quantum states under local stochastic dephasing channels can not pass through the axis, which indicates that quantum discord doesn’t have sudden death and sudden birth.

Another question is the quantity of quantum discord sudden birth. For any initial state, while *t* → ∞, there are *c*_1_(*t*_∞_) = *c*_2_(*t*_∞_) and *c*_3_(*t*_∞_) = 0. While *t* → ∞, $\frac{\partial {\mathscr{D}}}{\partial {c}_{1}}=0$ if and only if *c*_1_(*t*_∞_) = *c*_2_(*t*_∞_) = *c*_3_(*t*_∞_) = 0. Thus, the birth of quantum discord is maximized at *c*_1_(*t*_∞_) = 0.5, *c*_2_(*t*_∞_) = 0.5, *c*_3_(*t*_∞_) = 0 and *c*_1_(*t*_∞_) = −0.5, *c*_2_(*t*_∞_) = −0.5, *c*_3_(*t*_∞_) = 0. The maximum value of quantum discord sudden birth is 0.308, and the corresponding initial states are *c*_1_ = 1, *c*_2_ = *c*_3_ = 0 and *c*_1_ = −1, *c*_2_ = *c*_3_ = 0.

When we consider two kinds of local dephasing noises, we know that they compared with the collective quantum noises don’t have sudden death and sudden birth. We want to explore what makes them different, namely, what kind of noise channels can have this phenomenon for Bell-diagonal states. We might as well review the density matrix of Bell-diagonal states

33 $${\rho }_{AB}=(\begin{array}{cccc}1+{c}_{3} & 0 & 0 & {c}_{1}-{c}_{2}\\ 0 & 1-{c}_{3} & {c}_{1}+{c}_{2} & 0\\ 0 & {c}_{1}+{c}_{2} & 1-{c}_{3} & 0\\ {c}_{1}-{c}_{2} & 0 & 0 & 1+{c}_{3}\end{array}).$$

Obviously, the density matrix can be changed by quantum noisy channels through introducing a time-dependent coefficient in front of *c*_1_ + *c*_2_, *c*_1_ − *c*_2_, 1 − *c*_3_ and 1 + *c*_3_, which results in the change of quantum discord. Now, we assume that entire coefficients are exponential decay. A necessary condition of sudden death and sudden birth is that quantum discord becomes zero during its evolution and we have already known that quantum discord equals to zero if only if one of the parameters is limited to axes. So we need to consider three kinds of conditions. Firstly, parameters are limited to *c*_3_ axes. Considering the assumption, parameter *c*_3_ can’t reach zero for a finite time, so this situation is impossible. Then, parameters are limited to *c*_1_ or *c*_2_. In particular, both situations need the parameter *c*_3_ equals to zero, thus the initial value of *c*_3_ must be zero. Then, we obtain the first necessary condition that initial value of *c*_3_ must be zero.

Without loss of generality, *c*_1_(*t*) and *c*_2_(*t*) can be expressed by

34 $$\{\begin{array}{ccc}{c}_{1}(t)+{c}_{2}(t) & = & ({c}_{1}+{c}_{2}){e}^{-\alpha t},\\ {c}_{1}(t)-{c}_{2}(t) & = & ({c}_{1}-{c}_{2}){e}^{-\beta t},\end{array}$$

where *α* and *β* are positive real numbers. Equivalently,

35 $$\{\begin{array}{ccc}2{c}_{1}(t) & = & {c}_{1}({e}^{-\alpha t}+{e}^{-\beta t})+{c}_{2}({e}^{-\alpha t}-{e}^{-\beta t}),\\ 2{c}_{2}(t) & = & {c}_{1}({e}^{-\alpha t}-{e}^{-\beta t})+{c}_{2}({e}^{-\alpha t}+{e}^{-\beta t}).\end{array}$$

Next, we limit parameters to *c*_1_ axes, which signifies that the following equation is satisfied at finite time *t*_0_

36 $$\begin{array}{rcl}{c}_{1}({e}^{-\alpha {t}_{0}}+{e}^{-\beta {t}_{0}})+{c}_{2}({e}^{-\alpha {t}_{0}}-{e}^{-\beta {t}_{0}}) & = & \mu ,\\ {c}_{1}({e}^{-\alpha {t}_{0}}-{e}^{-\beta {t}_{0}})+{c}_{2}({e}^{-\alpha {t}_{0}}+{e}^{-\beta {t}_{0}}) & = & 0,\end{array}$$

where *μ* is a nonzero real number and satisfy −1 ≤ *μ* ≤ 1. We choose *c*_1_ and *c*_2_ as unknowns, it is not difficult to verify that the rank of the coefficient matrix always equals to the rank of augmented matrix so we can always find a set of *c*_1_ and *c*_2_, which make Eq. (36) true. Notably, *α* and *β* must be unequal, otherwise Eq. (36) can’t be true for finite time. In addition, the parameters limited to *c*_2_ axes can get the same conclusions with same methods. So the second necessary condition is that the coefficient of density matrix elements *c*_1_ + *c*_2_ and *c*_1_ − *c*_2_ must be unequal. ref.^36^ shows the local noise channels’ explicit expression and the coefficient of density matrix elements *c*_1_ + *c*_2_ and *c*_1_ − *c*_2_ are equal. It indicates that local quantum channels have no sudden death and sudden birth phenomenon. For example, the density matrix of Bell-diagonal states under a quantum noise channel can be expressed by:

37 $${\rho {\rm{^{\prime} }}}_{AB}(t)=(\begin{array}{cccc}1+{c}_{3} & 0 & 0 & {e}^{-0.2t}({c}_{1}-{c}_{2})\\ 0 & 1-{c}_{3} & {e}^{-t}({c}_{1}+{c}_{2}) & 0\\ 0 & {e}^{-t}({c}_{1}+{c}_{2}) & 1-{c}_{3} & 0\\ {e}^{-0.2t}({c}_{1}-{c}_{2}) & 0 & 0 & 1+{c}_{3}\end{array}).$$

**Figure 7:**
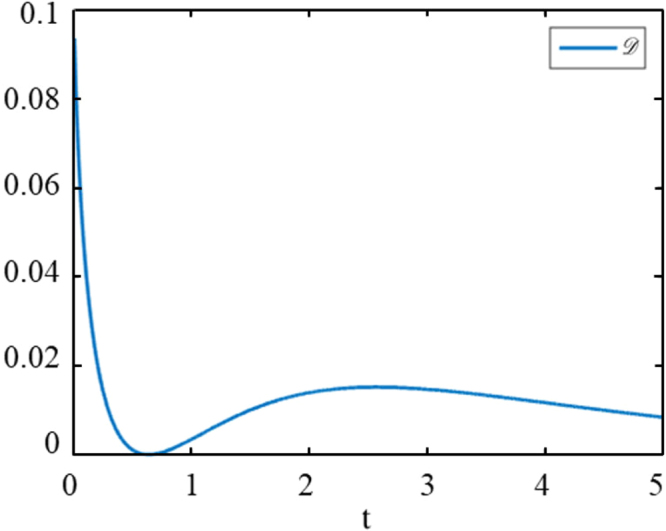
The *α* and *β* are 1 and 0.2, respectively, *c*_1_ = −0.8, *c*_2_ = 0.2, *c*_3_ = 0. Obviously, it satisfies *c*_3_ = 0 and *α* ≠ *β*. It’s worth noting that the two conditions are just necessary condition since initial states also play an important role.

Then, the quantum discord appears sudden death and birth, as shown in Fig. 7 below:

It is found that quantum discord is equal to coherence with an optimal basis. The relative entropy of coherence ${{\mathscr{C}}}_{re}({\rho }_{AB}^{{\sigma }_{i}})$ for Bell-diagonal states in computational bases *σ*_*i*_ are given by ref.^25^, where *σ*_*i*_ are Pauli matrixes with *i* = 1, 2, 3. The relation between quantum discord and coherence is intuitively characterized in Table 1, which signifies that the sudden death of quantum discord indicates the sudden death of quantum coherence in an optimal basis. The sudden death of coherence can appear in an optimal basis, if quantum discord has been sudden death. It implies that the robustness of coherence is stronger than quantum discord.

**Table 1:** For the Bell-diagonal states, quantum discord is equal to coherence with optimal basis.

Region	*c* = *c*_1_	*c* = *c*_2_	*c* = *c*_3_
Quantum discord	${{\mathscr{C}}}_{re}({\rho }_{AB}^{{\sigma }_{1}})$	${{\mathscr{C}}}_{re}({\rho }_{AB}^{{\sigma }_{2}})$	${{\mathscr{C}}}_{re}({\rho }_{AB}^{{\sigma }_{3}})$

## Conclusions

We have studied sudden death of quantum resources under three kinds of breaking channels, EBC, DBC and CBC. We introduce their unified expression called the Holevo form, which reveals the hierarchical relationship of breaking channels and their corresponding free states.

In addition, we have studied the dynamics of quantum discord under local and collective quantum noisy channels. The states we considered are Bell-diagonal states with explicit geometric structure in parameters space. Then sudden death and sudden birth of quantum discord is discovered under collective dephasing noise along the *z*-direction. We explain this interesting phenomenon and select the corresponding states with the help of geometry structure of Bell-diagonal states. Moreover, comparing the result of collective noise with local noise, we indicate that collective noise can increase quantum correlations of primary system, while local noise can not. Then we use two necessary conditions that *c*_3_ = 0 and *α* ≠ *β* to understand their differences and judge which quantum noise channels can make the Bell-diagonal states sudden death and birth.

Finally, according to the relationship between quantum discord and coherence, we indicate that the sudden death and sudden birth of quantum discord implies the sudden death and sudden birth of coherence in an optimal basis.
